# Supplementary material for: Real-world management, resource use, patient-reported outcomes and adherence in patients receiving direct oral anticoagulants for first stroke attributed to non-valvular atrial fibrillation in secondary care: A UK mixed-methods observational study
Source: PLoS One. 2025 May 23;20(5):e0321621. doi: 10.1371/journal.pone.0321621 (PMC12101689; doi:10.1371/journal.pone.0321621)
Supplement: S1 File — This document contains supplementary tables 1–7. (DOCX) [file pone.0321621.s001.docx]

**Supplementary materials for:**

**Real-world management, resource use, patient-reported outcomes and adherence in patients receiving direct oral anticoagulants for first stroke attributed to non-valvular atrial fibrillation in secondary care: a UK mixed-methods observational study**

James Uprichard, Liqun Zhang, Anand Dixit, Yaqoob Bhat, Amit Mistri, Dipankar Dutta, Khalid Rashed, Dumin Karunatilake, Chris Hatton, Joe Eva, Amelia Reed

**S1 Table. CHA2DS2-VASc risk scores for the 12 months prior to first stroke attributable to non-valvular atrial fibrillation.**

| **Demographic and clinical characteristics** | **Overall** | **Apixaban** | **Dabigatran** | **Dalteparin** | **Edoxaban** | **Enoxaparin** | **Rivaroxaban** | **Warfarin** | **None prescribed** |
| --- | --- | --- | --- | --- | --- | --- | --- | --- | --- |
| **CHA_2_DS_2_-VASc risk scores (2–9)*** | **n (%=224)** | **n (%=125)** | **n (%=3)** | **n (%=3)** | **n (%=62)** | **n (%=2)** | **n (%=6)** | **n (%=3)** | **n (%=20)** |
| 2 | 26 (12%) | 12 (10%) | 2 (67%) | 1 (33%) | 8 (13%) | 1 (50%) | 1 (17%) | 0 (0%) | 1 (5%) |
| 3 | 41 (18%) | 19 (15%) | 0 (0%) | 1 (33%) | 16 (26%) | 1 (50%) | 1 (17%) | 0 (0%) | 3 (15%) |
| 4 | 58 (26%) | 31 (25%) | 1 (33%) | 1 (33%) | 17 (27%) | 0 (0%) | 1 (17%) | 0 (0%) | 7 (35%) |
| 5 | 69 (31%) | 48 (38%) | 0 (0%) | 0 (0%) | 12 (19%) | 0 (0%) | 3 (50%) | 1 (33%) | 5 (25%) |
| 6 | 25 (11%) | 13 (10%) | 0 (0%) | 0 (0%) | 8 (13%) | 0 (0%) | 0 (0%) | 1 (33%) | 3 (15%) |
| 7 | 5 (2%) | 2 (2%) | 0 (0%) | 0 (0%) | 1 (2%) | 0 (0%) | 0 (0%) | 1 (33%) | 1 (5%) |
| *In accordance with the scoring criteria, prior stroke adds +2 to the CHA_2_DS_2_-VASc score. Since all participants in this study had had a prior stroke (as a function of the inclusion criteria for the study) the minimum possible score at index was 2. CHA2DS2-VASc scores were only calculated for those patients for whom all components of the score were available | | | | | | | | | |

**S2 Table. Recorded stroke severity - classification based on computed tomography (CT) scan**

| **Classification** | **n** | **% (n=94)** |
| --- | --- | --- |
| **Major** | 6 | 6% |
| **Minor** | 25 | 27% |
| **Not classified** | 35 | 37% |
| **Other** | 28 | 30% |
| **Missing** | 2 |  |

**S3 Table. Recorded stroke severity - clinician opinion of severity**

| **Classification** | **n** | **% (n=54)** |
| --- | --- | --- |
| **Mild** | 30 | 56% |
| **Moderate** | 16 | 30% |
| **Moderate to severe** | 5 | 9% |
| **Severe** | 2 | 4% |
| **Other** | 1 | 2% |

**S4 Table. Bespoke adherence questionnaire responses at 3- and 6-months post-stroke**

|  | **3 months** | **6 months** |
| --- | --- | --- |
|  | **n (%=68)** | **n (%=60)** |
| **Q1. What type of anticoagulant treatment are you currently taking?** | | |
| Apixaban (also known as Eliquis®) | 33 (49%) | 33 (55%) |
| Edoxaban (also known as Savaysa®, Lixiana®) | 29 (43%) | 22 (37%) |
| Not known | 1 (1%) | 1 (2%) |
| Rivaroxaban (also known as Xarelto®) | 5 (7%) | 4 (7%) |
| **Q2. How long have you been taking your current treatment?*** | | |
| 0–3 months | 38 (56%) | 4 (7%) |
| 3–6 months | 14 (21%) | 27 (46%) |
| 6–9 months | 0 (0%) | 14 (24%) |
| Over 3 months | 21 (31%) | 41 (69%) |
| Over 6 months | 0 (0%) | 18 (31%) |
| Other/not known | 9 (13%) | 10 (17%) |
| **Q3. Have you missed any dose of your current anticoagulation medication over the past 7 days?** | | |
| No | 65 (96%) | 60 (100%) |
| Yes | 3 (4%) | 0 (0%) |
| **Q3a. If 'Yes', what were the main reasons for this?** | | |
| Patient forgot | 2 (67%) | - |
| Nausea, tiredness and fatigue | 1 (33%) | - |
| **Q4. Have you previously received any other type of oral anticoagulant treatment (after your stroke)?** | | |
| No | 66 (97%) | 57 (95%) |
| Not sure | 2 (3%) | 2 (3%) |
| Yes | 0 (0%) | 1 (2%) |
| **Q4a – If 'Yes', what type of treatment did you previously receive?** | | |
| Aspirin | - | 1 (100%) |
| *Patients provided answers via free text, and these were then grouped into relevant time windows. Groups are not mutually exclusive. | | |

**S5 Table. Baseline care satisfaction responses**

**S6 Table. Precision of estimates of prevalence of risk factors**

| % | Number of patients | | | |
| --- | --- | --- | --- | --- |
|  | 250 | | 300 | |
|  | LCL | UCL | LCL | UCL |
| 5 | 2.3% | 7.7% | 2.5% | 7.5% |
| 10 | 6.3% | 13.7% | 6.6% | 13.4% |
| 15 | 10.6% | 19.4% | 11.0% | 19.0% |
| 20 | 15.0% | 25.0% | 15.5% | 24.5% |
| 25 | 19.6% | 30.4% | 20.1% | 29.9% |
| 30 | 24.3% | 35.7% | 24.8% | 35.2% |
| 35 | 29.1% | 40.9% | 29.6% | 40.4% |
| 40 | 33.9% | 46.1% | 34.4% | 45.6% |
| 45 | 38.8% | 51.2% | 39.4% | 50.6% |
| 50 | 43.8% | 56.2% | 44.3% | 55.7% |
| 55 | 48.8% | 61.2% | 49.4% | 60.6% |
| 60 | 53.9% | 66.1% | 54.4% | 65.6% |
| 65 | 59.1% | 70.9% | 59.6% | 70.4% |
| 70 | 64.3% | 75.7% | 64.8% | 75.2% |
| 75 | 69.6% | 80.4% | 70.1% | 79.9% |
| 80 | 75.0% | 85.0% | 75.5% | 84.5% |
| 85 | 80.6% | 89.4% | 81.0% | 89.0% |
| 90 | 86.3% | 93.7% | 86.6% | 93.4% |
| 95 | 92.3% | 97.7% | 92.5% | 97.5% |

**S7 Table. Precision of estimates of mean adherence score**

|  | | 40 patients | | 50 patients | |
| --- | --- | --- | --- | --- | --- |
| Expected MMAS-8 Score | SD | LCL | UCL | LCL | UCL |
| 7.2 | 1 | 6.9 | 7.5 | 6.9 | 7.5 |
|  | 1.2 | 6.8 | 7.6 | 6.9 | 7.5 |

LCL, lower 95% confidence limit; UCL, upper 95% confidence limit
