# Supplementary material for: Real-world management, resource use, patient-reported outcomes and adherence in patients receiving direct oral anticoagulants for first stroke attributed to non-valvular atrial fibrillation in secondary care: A UK mixed-methods observational study
Source: PLoS One. 2025 May 23;20(5):e0321621. doi: 10.1371/journal.pone.0321621 (PMC12101689; doi:10.1371/journal.pone.0321621)
Supplement: Attachment — Submitted filename: Response to reviewer comments_v1.0_20DEC2024.docx [file pone.0321621.s003.docx]

Response to reviewer comments:

We thank the reviewers for kindly spending the time to review this manuscript and providing their input. Below we have addressed the reviewers’ comments (Italicized below) and numbered them. Within the ‘marked’ copy of the revised manuscript, these changes can be found as tracked changes, in addition to being highlighted in yellow and with associated comments that contain the relevant comment number within this document.

**Reviewer 1:**

*Well presented manuscript regarding the use of anticoagulants in patients with stroke presenting real world data.
Adherence to the guidelines is important and it is highlighted on the current paper. Good quality of data.*

We thank the reviewer for their positive comments.

**Reviewer 2:**

*This is a well-written manuscript that describes a mixed-methods study investigating the use of direct oral anticoagulants (DOACs) in UK patients with a first stroke attributable to non-valvular atrial fibrillation (NVAF). The study provides valuable insights into patient characteristics, treatment patterns, adherence, and patient-reported outcomes.

Strengths:

The study clearly addresses the need for real-world data on DOAC use in stroke patients with NVAF. The study recruited a sufficient number of patients to draw meaningful conclusions which serves the owner of the study.

Weaknesses:

The study only followed patients for 6 months, which may not be sufficient to capture long-term adherence and outcomes.
It does not explore the reasons for the wide variation in time to DOAC initiation.: Not all patients completed all questionnaires, which could introduce bias.

Recommendations:*

1. *Abstract: The abstract could be strengthened by highlighting the key findings regarding adherence, satisfaction, and resource utilization*.

The key findings around adherence and satisfaction are already highlighted within the abstract. Specifically, that “At 3 and 6 months, 73% (46/63) and 83% (43/52) had high (score of 8) Morisky Medication Adherence Scale score (MMAS-8), respectively. No patients reported being dissatisfied at 3 or 6 months post-DOAC initiation.”

Regarding the addition of resource utilization results, we have added the following text (highlighted yellow below and in the tracked manuscript):

“For Group 2, the median time from stroke to first DOAC dose was 6 (IQR, 2.0–10.2; n=88) days; 50% patients had ≥1 outpatient visit recorded related to AF or DOACs.”

2. *Introduction: The authors could expand on the potential benefits and risks of DOAC use in this patient population.*

Text has been added to the introduction to better outline the potential benefits and risks of DOAC use in the studied patients as below:

“The increasing use of DOACs over vitamin K antagonists is likely reflective of their relative benefits including an improved efficacy:safety ratio, a favourable bleeding profile (although the risk of gastrointestinal bleeding is higher than that observed for warfarin ^1^), a reduced need for routine monitoring and fewer drug interactions ^2^.”

3. *Methods: The authors should clarify how missing data was handled in the analyses.*

It is specified in the section “Statistical analyses” that “Each analysis was only conducted from patients with available data in their medical records, with the total number of patients included specified alongside each result.” Missing data is common in real-world studies as there is no way to mandate all data to be collected in clinic, thus we accurately report the descriptive statistics for the data that has been collected and provide the relevant number of patients that it has been collected from.

4. *Results: The results section could be more concise by focusing on the most important findings. Consider presenting some data in tables or figures for better visualization.*

We thank the reviewer for their suggestions. However, we believe that the current draft of the results is appropriately concise given the granularity of the real-world data and that the current number of figures (1 results figure) and tables (2 tables) is pertinent to highlight those pieces of key information.

5. *Discussion: The discussion should elaborate on the potential reasons for delayed DOAC initiation and the implications for clinical practice.*

We thank the reviewer for this suggestion. There is already a paragraph on this in the discussion that outlines the observed levels of delay between stroke and DOAC initiation and reasons for delay/clinical implications. The paragraph is copied below for ease of review (and commented on in the manuscript).

“Whilst the median time between stroke and DOAC initiation was 6 days, it ranged from 0 days (indicating the DOAC was initiated on the day of the stroke) to 485 days post-stroke. The reason for the significant delay to DOAC initiation is not known and may relate to later increase in embolic risk and/or fall in bleeding risk. It is also possible that the larger values observed here relate to the use of subsequent Holter monitoring, although this was not specifically investigated within this study and publications evidence the importance of early investigations for AF and its subsequent impact on early anticoagulant prescriptions [35,36]. Guidance from the Royal College of Physicians recommends that anticoagulants are initiated within 2 weeks of ischaemic stroke for patients with known AF and no haemorrhagic transformation [37]. Observational data suggest that early anticoagulation initiation post-stroke may be beneficial in selected patients [38,39], however primary evidence to inform the appropriate time of initiation of anticoagulation post-stroke is lacking. Recent results from the ELAN trial suggest that early DOAC initiation (<48 hours after minor or moderate stroke, and on day 6 or 7 following major stroke) may be appropriate in acute stroke patients with NVAF [40]. Ongoing studies such as the OPTIMAS [41] are further exploring this issue. “

6. *Limitations: The limitations section should acknowledge the potential impact of the COVID-19 pandemic on resource utilization and patient follow-up.*

We thank the review for their suggestion. We have added the following statement to the limitations section in the discussion:

“Due to the overlap in time between the study and the COVID-19 pandemic, it should be noted that there may have also been an impact on the observed resource utilisation and subsequent patient follow-up.”

*Overall Recommendation:

This is a good quality study that deserves to be published in a peer-reviewed journal with minor revisions.*

We thank reviewer 2 for their comments and suggestions.

**Reviewer 3:**

*The authors present descriptive statistics of characteristics and outcomes of adult patients with nonvalvular atrial fibrillation (NVAF) receiving direct oral anticoagulants (DOACs) for secondary stroke prevention at secondary care center in United Kingdom (UK). Although this is interesting data, there are several issues which need to be clarified.*

7. *The title should include the fact that this study is based on real-world data from secondary care centers in UK*.

The title already contained the wording “real-world” but we have also now added in wording around the secondary care centres as highlighted below:

“Real-world management, resource use, patient-reported outcomes and adherence in patients receiving direct oral anticoagulants for first stroke attributed to non-valvular atrial fibrillation in secondary care: a UK mixed-methods observational study“

8. *As the author points out, it is important to identify AF before a stroke occurs. Considering that the minimum CHA2DS2vasc score is 2 points in group 1, the fact that anticoagulant therapy was not provided despite having the diagnosis of NVAF is a serious situation. The authors should describe the proportion of such patients and their background why they were not provided anticoagulation therapy despite having NVAF. It would also be desirable to show the proportion of patients who had already been receiving anticoagulant treatment for some reason but then developed a stroke*.

We thank the reviewer for their very important point.

Regarding the proportion of patients who had not been provided anticoagulants prior to stroke but had already received a diagnosis of non-valvular AF, we note that this is information of interest but that this was not an intended outcome of this study (please see the published study protocol)^3^. However, in order to provide additional information beyond the median (IQR) time from diagnosis of AF, we have additionally provided the range of days between diagnosis and stroke to evidence how widely this ranged (see below excerpt from Table 1 highlighted):

| Time from AF diagnosis to stroke (days, median [IQR; range])** | 0.0 (-1.0-0.0; -485.0-4,575.0) |
| --- | --- |

Regarding the proportion of patients who had already been receiving anticoagulant treatment, the study was designed with inclusion criteria to specifically recruit patients who had not received anticoagulants in the 12 months prior to their stroke and thus this data is not available from this dataset.

9. *Of the 93 registered patients in group 2, only 60 (64.5%) patients completed the 6-month bespoke adherence questionnaire. The authors should indicate the follow-up rate and DOACs prescription rate at 6 months. Since it is highly likely that DOACs are discontinued in patients who drop out, the authors should state the possibility of extremely low adherence in such cases.*

Regarding the follow-up rate and DOACs prescription rate at 6 months, this information is outlined in the section “Management pathway (Group 2)”. We state that of the 93 subjects in group 2, 86/93 patients had available information in their medical records about their DOAC treatment to be able to say whether they were or weren’t on treatment still at the end of the 6 months observation period. Of the 86 patients with available information, 85/86 (99%) were still being treated with their first DOAC at the end of the 6 months observation period; one patient was documented to have died within the 6 month observation period. This information is highlighted and commented for reference in the marked up manuscript.

Regarding the completion rate of the adherence questionnaires, it is common in real-world observational studies for subject numbers to drop-off over time. Although it should be noted firstly that at the first time point of 3 months, 68 patients complete the questionnaire suggesting that of those that completed the questionnaire initially, a high percentage (60/68, 88%) return the questionnaire at the latter time point.

Lastly, given that the recorded levels of DOAC discontinuation (within the 6 month observation period) are very low for those patients whom had data available, the numbers responding to the questionnaire are not indicative or related to the numbers of patients maintaining treatment and thus we suggest that there is no need to state the possibility of low adherence in these cases.

10. *The author should clearly describe the method of obtaining consent in Methods section of the manuscript. In particular, the author should clearly state whether consent was obtained from all participants, including Group 1.*

We have added the following highlighted text to the methods section in order to provide further clarity over consent:

“Patients were contacted and recruited by members of their direct care team between 23 January 2019 and 30 June 2021; recruitment was paused between April 2020 to June 2020 due to the COVID-19 pandemic. All patients gave informed consent to take part in the study.”

11. *The authors should clearly state the inclusion and exclusion criteria for this study in Methods section of the manuscript*.

We thank the reviewer for raising this important point. The inclusion/exclusion criteria are laid out in the protocol manuscript but have now been reiterated in this publication text. The following text has been added into the methods section as highlighted below:

“All patients were eligible for inclusion in the study if they presented to the study centre with a first ischaemic stroke which was, in the clinician’s opinion, attributable to NVAF and were aged ≥18 years at the time of first stroke. In addition, to have been eligible for Group 2, patients in Group 1 must have been initiated on apixaban, edoxaban or rivaroxaban after their first stroke. All patients were excluded from participation in the study if they were unwilling or unable to give written informed consent, if their medical records were not available for review, if they had been prescribed anticoagulants for any purpose in the 12 months prior to the stroke diagnosis, if they had prior or current haemorrhagic stroke, if they had a diagnosis of transient ischaemic attack, and/or if they had a severe cognitive or emotional deficit. Patients from Group 1 were excluded from Group 2 if they were unwilling or unable to complete the patient-reported questionnaires.”

12. *Because the patients who have stroke occurring despite anticoagulant treatment being provided are considered clinically important, the author should clearly state the rational for excluding them.*

This criteria was agreed upon at the period of study design as there was emerging real-world data around the use of DOACs in patients with AF, however there was a clear knowledge gap at the time around the use of DOACs in patients at the time of first ischaemic stroke.^3^ Thus, whilst we acknowledge the clinical importance of these patients, the study was designed to fill a different gap in clinical knowledge.

13. *The absence of patients who is treated by pulmonary vein isolation (PVI) is unnatural in recent era. If the PVI is to be excluded, the author should clearly state PVI as an exclusion criterion. In that case, the author should clearly state the background why PVI is not indicated.*

We thank the reviewer for raising this important point. No ablation was recorded as treatment in the patients included in this study, this is likely because the majority of patients had their AF diagnosed on (or around) the date of their index stroke and subsequently, as per current NICE guidance in the UK, were started on anticoagulants in the first instance for secondary stroke prevention.^4^ NICE guidance currently states the use of catheter ablation (as PVI is more commonly termed in the UK) is an option if “drug treatment is unsuccessful, unsuitable or not tolerated” meaning that it is likely that any patients that required ablation, may have received this treatment after the study observation period as the majority of patients with data available (99%, n=85/86) remained on their first prescribed DOAC treatment at the end of the study observation period.^4^

Ablation was not part of the study exclusion criteria, nor was it specifically not recorded. We strongly feel that this subsequently does not warrant additional text in the manuscript introductory section as it is not the focus of the study and manuscript.

14. *The authors should clearly state the rational reason for excluding dabigatran in group 2. In addition, the author should add the demographics of group 2*.

The study focused on the use of direct anti-FXa inhibitors whilst dabigatran is a thrombin (factor IIa) inhibitor as written in the concluding paragraph of the introduction. Thus, dabigatran was not included in Group 2.

As per the study design laid out in the published study protocol,^3^ the demographics for Group 2 are not provided separately to Group 1.

15. *The creatinine clearance (Ccr) value is very important when prescribing DOACs. Therefore, the creatinine clearance value at the beginning of DOACs and at the end of the observation period should be added. Similarly, the author is encouraged to add the percentage of patients who were prescribed DOACs at inappropriate doses.*

We agree with the reviewer that CCr is an important measure when prescribing DOACs. Unfortunately, only a very small number of patients had baseline information available in their medical records (14 of 234) and thus it was decided not to include this information in the manuscript as it could be heavily biased due to its small sample size. Additionally, due to the way the data was recorded for the post-DOAC observation period, only 4 patients’ data is available and it is only recorded as an average CCr value.

Regarding the dosing, this information is provided in the “Management pathway (Group 2) section” of the manuscript (copied below for reference) which outlines that 80% or more of the patients (per DOAC subgroup) were prescribed the DOAC at the recommended dosage. We also realised that n=1 patients dosing was not written in for the apixaban group and this has been added to the manuscript and text below (highlighted yellow):

“Of those patients prescribed apixaban, 87% (n=40/46) of prescriptions were 5.0 mg twice daily, 11% (n=5/46) were 2.5 mg twice daily, 2% (n=1/46) were 15.0 mg twice daily; for edoxaban, 89% (n=33/37) of prescriptions were 60.0 mg once a day, 11% (n=4/37) were 30.0 mg once a day; for rivaroxaban, 80% (n=4/5) of prescriptions were 20.0 mg once a day and 20% (n=1/5) were 15.0 mg once a day”.

Given the real-world nature of this data, we have clearly outlined the data we were able to extract from records regarding dosing. We are however unable to link the specific doses of first DOAC prescribed to the CCr values due to the low sample size/availability from medical records. We have also added the following text to the limitations section on this point:

“We were unable to link the initially prescribed doses of DOACs to creatinine clearance data at baseline due to a low number of records being available which may introduce bias due to small sample size (<15 records available).”

16. *The author should state the basis for the sample size setting in Statistical analyses of Methods section. In addition, the author should also state that this study did not reach*

As this was an observational study with no planned comparative analyses, the sample size was calculated using precision estimates. Text has been added to the methods and the supplementary material to support this as evidenced below:

“As the study was planned to be descriptive, the study sample size was based upon precision estimates (95% confidence interval [CI]). No comparative analyses were performed. Precision estimates were performed based on: expected proportions of patients demonstrating certain characteristics, for example showing that with a sample of n=250, of which 20% are shown to have vascular disease, the confidence limits are between 15%-25%, suggesting reliable estimates will be obtained with this sample size (Group 1; Supplementary Table 6), for precision around the use of the MMAS-8 assuming a mean adherence score of 7.2 (based upon previously published work ^5^), a sample of 40 patients was observed to give lower and upper confidence limits of 6.8 and 7.6 (standard deviation - 1.2) suggesting reliable estimates will be obtained with this sample size (Group 1; Supplementary Table 7).”

17. *In Table 1, the 10 cases that could not be calculated CHA2DS2casc score should be included in the table 1.*

Table 1 includes the data for all patients where it was available. However, to further clarify why 10 patients fewer had the score calculated the following text was added to the table legend:

“(10 patients had missing data and thus did not have their score calculated).”

18. *The definition of body mass index (BMI) categories should be described in Methods section.*

The following text has been added (in addition to the current text description in the table legend):

“Body mass index (BMI) categories were calculated based upon numerical thresholds: underweight < 18.5, healthy weight: ≥ 18.5 to < 25, overweight ≥ 25 to < 30, obesity ≥ 30.”

19. *The definition of renal impairment should be clearly stated in Methods section. In particular, it should be clearly stated whether the definition includes urinary protein or is based solely on eGFR. In addition, since the DOAC dosage is set based on Ccr, Ccr should also be stated*.

History of renal impairment was taken from medical records such that any records of it are reflective of it being written down in the notes. We are thus unable to specifically qualify how the clinician defined it. The following statement has been added to the Table 1 legend for clarity:

“ **Renal impairment and alcohol use (yes/no) was recorded as defined in medical records.”

20. *The definition of alcohol consumption should be clearly stated in Methods section. Some papers have suggested a link between heavy drinking and AF. It should be clearly stated whether this includes only heavy drinkers or also occasional drinkers who drink less than twice a week.*

Similar to the renal impairment, question above, the question regarding alcohol use was a yes/no answer that reflected whether alcohol use was recorded in medical notes. An additional note has been added as below to the Table 1 legend:

“ **Renal impairment and alcohol use (yes/no) was recorded as defined in medical records.”

21. *The author should add the study flow diagram of this study. In particular, since the population differs for hospital care resource utilisation, MMAS-8 scale, and TSQM-9, it is desirable to include the population parameters for the available data in the diagram.*

We thank the reviewer for their suggestion but we would argue that this is not required. Given that a study flow diagram would usually outline the overall numbers of participants in the overall and sub-cohorts whereas the reviewer is talking about how many participants completed questionnaires or had resource use data available from medical records. It is common practice for real-world observational studies to accurately account for how many participants had the relevant data or variable available and to account for this (i.e. provide the total number of participants providing this) and this is how this manuscript has been written.

22. *It is stated that 86 people completed the scheduled observation period for group 2. The details of the 7 people who dropped out should be clearly stated in the Result section and study flow diagram*.

We assume that the reviewer is referring to the n=86 of the following statement:

“Of 86 patients with available information in their medical records, 99% (n=85) patients were still being treated with their first DOAC at the end of the study observation period; one patient was documented to have died at 3.8 months”

This is a limitation of real-world data in which we are limited by the records kept (as we cannot mandate the recording of specific data). Thus we only have 86 patients with this data recorded, but this does not mean that they did not complete the observation period. As with the previous comment (comment 21), we argue that the use of a study flow diagram is not required and/or relevant as we can only report the data that was collected.

23. *The use of unnecessary and uncommon abbreviations should be discouraged.*

We thank the reviewer for their comment, however, we believe that the abbreviations utilised within the manuscript are relevant and appropriate to the topic and audience.

References

1.	Scridon A, Balan AI. Challenges of Anticoagulant Therapy in Atrial Fibrillation—Focus on Gastrointestinal Bleeding. Int J Mol Sci. 2023 Apr 7;24(8):6879.

2.	Shen NN, Zhang C, Wang N, Wang JL, Gu ZC, Han H. Effectiveness and Safety of Under or Over-dosing of Direct Oral Anticoagulants in Atrial Fibrillation: A Systematic Review and Meta-analysis of 148909 Patients From 10 Real-World Studies. Front Pharmacol [Internet]. 2021 Mar 18 [cited 2024 Nov 13];12. Available from: https://www.frontiersin.org/journals/pharmacology/articles/10.3389/fphar.2021.645479/full

3.	Bhat Y, Dixit A, Mistri A, Patel B, Quoraishi SH, Uprichard J. A mixed methodology, non-interventional study to evaluate the use of direct oral anticoagulants in UK clinical practice for patients with a first stroke associated with non-valvular atrial fibrillation: study protocol. BMC Neurology. 2019 Nov 29;19(1):306.

4.	National Institute for Health and Care Excellence. Atrial fibrillation: diagnosis and management. Atrial fibrillation [Internet]. 2021; Available from: https://www.nice.org.uk/guidance/ng196

5.	Stephenson JJ, Shinde MU, Kwong WJ, Fu AC, Tan H, Weintraub WS. Comparison of claims vs patient-reported adherence measures and associated outcomes among patients with nonvalvular atrial fibrillation using oral anticoagulant therapy. Patient Prefer Adherence. 2018 Jan 12;12:105–17.
