# Supplementary material for: Real-world management, resource use, patient-reported outcomes and adherence in patients receiving direct oral anticoagulants for first stroke attributed to non-valvular atrial fibrillation in secondary care: A UK mixed-methods observational study
Source: PLoS One. 2025 May 23;20(5):e0321621. doi: 10.1371/journal.pone.0321621 (PMC12101689; doi:10.1371/journal.pone.0321621)
Supplement: Attachment — Submitted filename: Response to reviewer comments_R1_v1.0_24FEB2025.docx [file pone.0321621.s004.docx]

Response to reviewer comments R1:

We thank the reviewers for kindly spending the time to review this manuscript and providing their input. Below we have addressed the reviewers’ comments (Italicized below) and numbered them. Within the ‘marked’ copy of the revised manuscript, these changes can be found as tracked changes, in addition to being highlighted in yellow and with associated comments that contain the relevant comment number within this document.

**Reviewer 1:**

**Comment**

*Very well presented manuscript.*

*Given the inclusion of the review comments it is suitable for publication*

**Response**

We thank the reviewer for their positive comments.

**Reviewer 2:**

**Comment**

*This study investigates the use of direct oral anticoagulants (DOACs) in adult patients with non-valvular atrial fibrillation (NVAF) who had a first ischaemic stroke. The study included 234 patients in Group 1 and 93 in Group 2. Group 2 patients were initiated on apixaban, edoxaban, or rivaroxaban post-first stroke. The primary objective was to describe patients' demographics, clinical characteristics, and medical history, stratified by the anticoagulant prescribed. The secondary objectives were to describe patient management pathways, hospital resource use, clinical assessments associated with DOAC treatment, and patient-reported satisfaction and experience of DOAC treatment.*

*The results demonstrate high levels of adherence, persistence, and treatment satisfaction in the 6 months post-initiation of DOAC after first stroke attributable to NVAF. Patients were largely initiated on DOACs in compliance with guidelines, with most patients prescribed DOACs at the suggested dosages and very low rates of dose changes or interruptions over the post-index observation period. Patient-reported satisfaction with DOAC treatment was positive, with no patients reporting being dissatisfied at either the 3- or 6-month time point post-DOAC initiation.*

*The study found that the median time between stroke and DOAC initiation was 6 days, ranging from 0 days to 485 days post-stroke. The most common reason given for DOAC choice was clinical opinion. The majority of patients observed received the recommended doses of apixaban, edoxaban and rivaroxaban as per UK guidelines, with high levels of treatment persistence with the prescribed DOAC.*

*Regarding hospital care resource utilization, only 50% of patients had 1 or more outpatient clinic appointments recorded in the post-DOAC observation period. Adherence to DOACs as measured by the MMAS-8 was broadly consistent across the 3- and 6-month time points post-initiation.*

*The study's limitations include a smaller sample size than planned and potential impacts on observed resource utilization and subsequent patient follow-up due to the overlap in time between the study and the COVID-19 pandemic.*

*In conclusion, this study provides real-world evidence of the patient characteristics, management pathways, and patient-reported experiences of patients prescribed a direct FXa inhibitor DOAC after first stroke attributable to NVAF in UK clinical practice. The results provide clinicians with valuable insights into the experience of post-stroke patients with NVAF receiving direct FXa inhibitor DOACs treatment for secondary prevention of stroke.*

**Response**

We thank reviewer 2 for their comments.

**Reviewer 3:**

**Comment**

*1. The authors have specifically indicated that a group of patients who have been diagnosed with non-valvular atrial fibrillation (NVAF) but are not receiving anticoagulation therapy are included in this cohort. Unfortunately, demographic information for this group of patients in the cohort is not available because it was not an intended outcome of this study. I recommend to include in the Limitations section that demographic information for this group of patients with known AF prior to stroke who were not anticoagulated, including those with a CHA2DS2 VASc Risk Score of 2 or more, was not available because it was not an intended outcome of this study. In addition, please note the following points: (1) In the Methods section, Group 1 is conoised of “patients with known AF prior to stroke who were not anticoagulated” and “those presenting with stroke who were found to have AF at the time of presentation or after”; and (2) The primary objective of this paper is stated as “to describe the demographics, clinical characteristics and medical history...”.*

**Response**

Regarding the demographic information for those patients diagnosed with NVAF but not receiving anticoagulation, the following text has been added to the limitations section to reflect that this subgroup was not specified as an outcome and that this may be of interest for future research (pg 15) as suggested:

As it was not a predefined outcome, demographics were not specifically provided for those patients in Group 1 who were diagnosed with NVAF but had not received anticoagulation therapy prior to first stroke, however future research should attend to these patients.

**Comment**

*2. As the study flow diagram is not used, the author should describe the population of the statistics in more detail when describing each statistic. For instance, for the 86 patients, there is only a statement that “Of 86 patients with available information in their medical records.” If the patients are a group for whom medical records are available at 6 months, this should be stated as such. Similarly, I recommend that the author states what the population of 80 patients is in the HCRU and clinical assessments. I also suggest that the author may comment on the seven patients without medical records in the Limitations section.*

**Response**

The text has been edited for greater clarity as highlighted below (pg 10):

“Of the patients in Group 2 with available information in their medical records (n=86/93), 99% (n=85/86) were still being treated with their first DOAC at the end of the study observation period; one patient was documented to have died at 3.8 months.”

“Of the 93 patients in Group 2 with relevant available data in their medical records (n=80/93), half (50%, n=40/80; n=13 had no data recorded) had at least one outpatient visit within the post-DOAC observation period related to AF or DOACs….”

The text has also been edited to refer to the limitation of being reliant on the completeness of medical records (pg 16):

“Real-world data from medical records and patient-reported measures rely on the completeness of medical records and/or the given answers; these were not queried or followed up to clarify inconsistencies or complete missing answers”

**Comment**

*3. The comment in the previous round means that the author is encouraged to state the rational reason for excluding dabigatran in Group 2, i.e. the reason for focusing on anti-FXa direct oral anticoagulants (DOACs). For instance, I suggest that the author may use descriptions such as “There are also limited data related to use of anti-FXa DOACs in routine clinical practice” from the protocol paper. Similarly, as with the protocol paper, I propose that DOACs be described as anti-FXa DOACs.*

**Response**

This has been addressed throughout the manuscript utilizing the term “FXa-inhibiting DOACs” in order to be more specific to the mechanism of the drugs. Though it should be noted as Group 1 data included a range of DOACs (not only FXa-inhibiting DOACs) that the text only refers to FXa-inhibiting DOAC(s) where pertinent.

**Comment**

*4. PACI, LACI, POCI, and TACI are not common abbreviations for non-neurologists, and as they only appear once in this paper, it is considered to be unnecessary to include as the abbreviations. Please consider deleting them.*

**Response**

These abbreviations are the ones recorded from the medical records; thus they have been kept in the manuscript to reflect this. However, to improve clarity for the broader readership of the journal, the abbreviations have been moved into parentheses and the full text versions moved into the main text.

“…documented as having had partial anterior circulation infarcts (PACI), 22% (n=26) as having lacunar infarcts (LACI), 12% (n=14) as having posterior circulation infarcts (POCI) and 8% (n=9) recorded total anterior circulation infarcts (TACI).“

References

1.	Scridon A, Balan AI. Challenges of Anticoagulant Therapy in Atrial Fibrillation—Focus on Gastrointestinal Bleeding. Int J Mol Sci. 2023 Apr 7;24(8):6879.

2.	Shen NN, Zhang C, Wang N, Wang JL, Gu ZC, Han H. Effectiveness and Safety of Under or Over-dosing of Direct Oral Anticoagulants in Atrial Fibrillation: A Systematic Review and Meta-analysis of 148909 Patients From 10 Real-World Studies. Front Pharmacol [Internet]. 2021 Mar 18 [cited 2024 Nov 13];12. Available from: https://www.frontiersin.org/journals/pharmacology/articles/10.3389/fphar.2021.645479/full

3.	Bhat Y, Dixit A, Mistri A, Patel B, Quoraishi SH, Uprichard J. A mixed methodology, non-interventional study to evaluate the use of direct oral anticoagulants in UK clinical practice for patients with a first stroke associated with non-valvular atrial fibrillation: study protocol. BMC Neurology. 2019 Nov 29;19(1):306.

4.	National Institute for Health and Care Excellence. Atrial fibrillation: diagnosis and management. Atrial fibrillation [Internet]. 2021; Available from: https://www.nice.org.uk/guidance/ng196

5.	Stephenson JJ, Shinde MU, Kwong WJ, Fu AC, Tan H, Weintraub WS. Comparison of claims vs patient-reported adherence measures and associated outcomes among patients with nonvalvular atrial fibrillation using oral anticoagulant therapy. Patient Prefer Adherence. 2018 Jan 12;12:105–17.
